# A comparison of the effects of physical and chemical mutagens in sesame (*Sesamum indicum* L.)

**DOI:** 10.1590/S1415-47572010005000090

**Published:** 2010-12-01

**Authors:** Tamina Begum, Tapash Dasgupta

**Affiliations:** Department of Genetics and Plant Breeding, Institute of Agricultural Science, University of Calcutta, KolkataIndia

**Keywords:** effectiveness, efficiency, mutagens, quantitative characters, sesame

## Abstract

Three sesame genotypes (Rama, SI 1666 and IC 21706) were treated with physical (γ-rays: 200 Gy, 400 Gy or 600 Gy) or chemical (ethyl methane sulphonate, EMS: 0.5%, 1.0%, 1.5% or 2.0%) mutagens and their mutagenic effectiveness and efficiency were estimated in the M _2_ generation. The M _3_ generation was used to identify the most effective mutagen and dose for induction of mutations. The average effectiveness of EMS was much higher than γ-rays. The lowest dose of γ-rays (200 Gy) and the lowest concentration of EMS (0.5%) showed the highest mutagenic efficiency in all genotypes. Analysis of the M _3_ generation data based on parameters such as the variance ratio and the difference in residual variances derived from the model of Montalván and Ando indicated that 0.5% concentration of EMS was the most effective treatment for inducing mutations.

The usefulness of any mutagen in plant breeding depends not only on its mutagenic effectiveness, but also on its mutagenic efficiency, efficient mutagenesis being the product of the maximum desirable changes accompanied by the least possible undesirable changes. Effectiveness and efficiency are two distinct properties of mutagens that have been extensively discussed elsewhere (Kawai, 1975, 1986; Shah *et al.*, 2008; Girija and Dhanavel, 2009). While ionizing radiation still remains the most suitable means for inducing variability (Brunner, 1995; Bhatia *et al.*, 2001; Irfaq and Nawab, 2003; Joseph *et al.*, 2004; Sangsiri *et al.*, 2005; Tah, 2006) a number of chemicals have been found to be equally and even many times more effective and efficient mutagens (Thakur and Sethi, 1995; Kharkwal, 1998; Solanki, 2005; Rekha and Langer, 2007; Basu *et al.*, 2008, Dhanavel *et al.*, 2008; Ganapathy *et al.*, 2008; Wani, 2009). Effectiveness usually means the rate of point mutations relative to dose, whereas efficiency refers to the rate of point mutations relative to other biological effects induced by the mutagen and is considered a measure of damage (Konzak *et al.*, 1965). Thus, two agents may be equal in mutagenic effectiveness because, at a given dose, they induce a mutation with the same frequency. However, when they diverge in their ability to produce undesirable changes such as sterility and lethality then they may be said to differ in mutagenic efficiency.

It is common practice to use only normal-looking M_2_ plants to obtain the M_3_ generation and apply preliminary visual selection in M_3_. This necessarily results in an increase in the volume of materials to be pursued and delays the isolation of promising variants. Consequently, considerable effort has been devoted to explore the possibility of selecting mutants with polygenic variability right from M_2_, which is the first generation to express variability after treatment. Moreover, the selection of promising variants in an early generation allows the rejection of unmutated “roughage” in M_2_. Obviously, mutagenic treatment is the sole cause of increasing variability in M_2_, especially considering that the check variety does not exhibit such variability. Further segregation of the polygenic system in M_3_ is expressed as the release of additional intrinsic variability. This being the mechanism of induction and inheritance of micromutations, selection in M_2_ or M_3_ can certainly help in identifying progenies that are likely to evince greater variability and a better response to selection.

The choice of effective mutagen and dose to be used (preferably one that induces enormous variability in any crop) is an important step. Montalván and Ando (2005) proposed a model to assess the effects of treatment on the mean and variance of mutagen-treated plants. Such an assessment could provide useful information for future sesame breeding programs.

In the present investigation, we examined the mutagenic effectiveness and efficiency of different doses of gamma (γ)-radiation and ethyl methane sulphonate (EMS) on the genetic variability of sesame in the M_2_ and M_3_ generations. The model of Montalván and Ando (2005) was applied to the M_3_ generation to identify the most effective mutagen and the dose that induced mutations.

Three sesame (*Sesamum indicum* L*.*) genotypes (Rama, SI 1666 and IC 21706) were selected for this study. One physical mutagen (gamma- or γ-rays) and a chemical mutagen (EMS) were used to induce mutations. Dry seeds (10%-12% moisture content) of each genotype were exposed to one of three doses (200 Gy, 400 Gy and 600 Gy) of γ-rays derived from ^60^Co. Irradiation was done at the rate of 30 Gy/min in the gamma garden of the Central Research Institute for Jute and Allied Fibres (CRIJAF) at Kolkata, West Bengal, India. Three hundred seeds per genotype were treated with each dose of radiation. In the case of EMS, seeds (150 per concentration) were exposed to one of four concentrations of the chemical (0.5%, 1.0%, 1.5% and 2.0% in phosphate buffer of neutral pH) for 6 h with intermittent shaking. The treated seeds were then washed with water, dried with filter paper and immediately sown in the field. By the end of the experiments, each genotype had been subjected to eight treatments (control, three doses of γ-rays and four concentrations of EMS).

The seeds were sown at the end of February, 2004 at the Agricultural Experimental Farm of the University of Calcutta. The resulting plants were designated as M_1_ plants and subsequently M_2_ and M_3_ plants were grown. The spacing between rows and plants was 35 cm and 10 cm, respectively. A randomized block design was followed with three replications. Normal cultural practices were followed during the entire growing period. Mutagenic effectiveness and efficiency were estimated based on the relative frequency of family segregation in the M_2_ generation, as described by Konzak *et al.* (1965). Ten families were selected for the M_3_ generation based on their means. In M_3_, ten progeny rows were raised with a single control row for each treatment. The seeds were sown in the fourth week of February, 2006 in a randomized block design with three replications. The yield and yield components, *i.e.*, plant height (cm), number of branches per plant, number of capsules per plant, number of seeds per capsule, 1000-seed weight (g) and seed yield per plant (g) in the M_3_ generation were recorded.

The mean value, standard deviation, variance and coefficient of variation (CV) for each trait were estimated. The relative CV was estimated based on the CV of the mutagen-treated population (CVt) and the non-treated control sample (CVnt), according to the method of Montalván and Ando (2005). The F-test was used to determine the significance of the increase in genetic variance attributed to mutagenic treatment, where



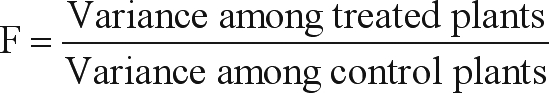


According to Ando and Vencovsky (1967), the difference between the residual variances of the treated and control samples provides an estimate of the increase in variance attributable to treatment with the mutagen and was computed as:

Dt = sr^2^ - so^2^

where Dt is the increase in variance attributable to treatment with the mutagen, sr^2^ is the residual variance resulting from treatment with the mutagen and so^2^ is the residual variance of the control.

The results for the M_2_ generation revealed that lower doses of mutagens were effective and efficient in causing polygenic variability in various quantitative characters, with a negative relationship between effectiveness and mutagen dose. These findings agreed with those of Roy Chowdhury *et al.* (2004) in mungbean, Dhanavel *et al.* (2008) in cowpea, Ganapathy *et al.* (2008) in little millet, and Sharma *et al.* (2005) and Thilagavathi and Mullainathan (2009) in black gram. The lowest concentration of EMS (0.5%) was the most effective in causing mutations. The average effectiveness of EMS was several times higher than γ-rays. Among the genotypes, 0.5% EMS was most effective in SI 1666 compared to Rama and IC 21706. Interestingly, γ-rays were more efficient in inducing mutations, with a lower dose of γ-rays showing a higher mutagenic efficiency, regardless of the genotypes. Studies in wheat (Gaul and Aastveit, 1966), *Arabidopsis thaliana* (Brock, 1971) and cowpea (Girija and Dhanavel, 2009) have also shown that EMS is more effective than radiation in inducing polygenic variability. On the other hand, various studies have shown that ionizing radiation induces greater polygenic variability than chemical mutagens (Murty and Oropeza, 1989; Sorour *et al.*, 1999; Larik *et al.*, 2009) and is therefore a powerful tool for engendering such variability. The earlier findings are therefore inconclusive.

Ionizing radiations such as γ-rays are highly effective in inducing chromosomal aberrations (Nilan and Konzak, 1961) whereas mutagens such as EMS act primarily on base pairs of the DNA molecule and yield a higher number of gene mutations. Because of the basic mechanistic difference between these two groups of mutagens, chemical mutagens are generally considered to be superior to physical mutagens for induction of mutation (Nilan and Konzak, 1961). Our findings for the M_2_ generation generally agreed with this conclusion. The M_3_ generation was studied further to affirm the conclusion of M_2_ generation.

Analysis of the results for the M_3_ generation using the model of Montalván and Ando (2005) revealed important changes in the mean values of various traits in the treated groups of the three genotypes when compared to the controls. The highest percentage change caused by γ-rays (244.16%) was observed in the number of branches per plant for SI 1666 seeds irradiated with 200 Gy, whereas 0.5% EMS recorded maximum percentage change (207.78%) for the number of capsules per plant derived from IC 21706 seeds (Table 1). The CVt/CVnt ratio indicated that the γ-ray dose of 200 Gy was most effective in affecting the seed yield per plant and most of the yield components in irradiated population of Rama. On the other hand, a dose of 400 Gy was more effective in influencing the number of capsules per plant, 1000-seed weight and seed yield per plant in SI 1666 and IC 21706 (Table 1). Interestingly, the most effective EMS concentration varied among mutant populations, as indicated by the CVt/CVnt ratio. Thus, 0.5% concentration of EMS was most effective in influencing the number of branches per plant, number of capsules per plant and seed yield per plant in all genotypes, whereas a concentration of 2.0% had a greater effect on plant height, number of seeds per capsule and 1000-seed weight in Rama plants; concentrations of 1.5% and 1.0% were more effective on these three parameters in SI 1666 and IC 21706 plants, respectively (Table 1).

There was a decrease in the coefficient of variation (CV) for all of the characters when data from individual plants (Table 1) rather than average data (Table 2) were considered. The highest CV (and correspondingly high standard error) was recorded for the number of branches per plant in SI 1666 plants exposed to 0.5% EMS. Interestingly, the relative CV for the number of capsules per plant and number of seeds per capsule in mutant population of Rama, the number of branches per plant in mutants of SI 1666, and plant height, number of branches per plant, number of capsules per plant, number of seeds per capsule and seed yield per plant in mutant population of IC 21706 was > 1.0, indicating an increase in variance with these treatments (Table 1). The degree of variation in the number of capsules per plant was remarkably high in mutants of the three genotypes, but was low for 1000-seed weight (Table 1). Among the different treatments, 0.5% EMS consistently produced greater variation in all of the characters except for plant height in Rama and SI 1666.

The increase in variance in the treated populations (Table 3) was an important indicator of the efficiency of the mutagen in inducing genetic variability. The F-test results showed that the mutagens were efficiently increased the variances of all of the traits except for 1000-seed weight (Table 3). The variance ratio (Vt/Vnt) was higher for all characters in plants from Rama seeds irradiated with 400 Gy and 600 Gy, whereas plants from SI 1666 and IC 21706 seeds exposed to 200 Gy and 600 Gy had higher F-variances for seed yield and many of the yield components. On the other hand, 0.5% EMS produced higher F-variances for the number of capsules per plant and seed yield per plant, regardless of the genotypes (Table 3). These findings further confirmed that these doses were more effective in producing variability. In general, the variance ratio was lower for 1000-seed weight when compared to other characters, regardless of the treatment.

A combined analysis of the different parameters, as proposed by Montalván and Ando (2005), suggested that 0.5% EMS was the best mutagenic treatment since it produced the greatest variability. This conclusion is drawn from the results regarding the effectiveness and efficiency of this concentration in the M_2_ generation. Overall, the results of this study indicate that the use of lower doses of chemical mutagens (0.5% in the case of EMS) that do not cause drastic chromosomal damage may be more effective in increasing the amount of variability.

## Figures and Tables

**Table 1:** Analysis of variance for six characters in the M_3_ generation.

Source of variation	df	Plant height	Number of branches/plant	Number of capsules/plant	Number of seeds/capsule	1000-seed weight	Seed yield/plant
Replication	1	0.21	0.54	2.76	0.18	0.01	0.10
Variety	2	24597.62**	77.44**	10464.08**	300.69**	0.10**	386.04**
Treatment	7	4208.71**	174.93**	9668.57**	186.19**	0.10**	337.77**
Variety x Treatment	14	2458.54**	48.32**	9412.63**	103.50**	0.14**	386.37**
Line	9	404.30**	22.55**	1158.82**	22.96**	0.06**	45.84**
Variety x Line	18	295.70**	32.18**	892.02**	20.80**	0.03**	33.29**
Treatment x Line	63	446.29**	25.71**	2205.39**	31.35**	0.07**	88.10**
Variety x Treatment x Line	126	511.79**	27.13**	2275.71**	45.16**	0.05**	84.86**
Errors	239	1.19	0.65	4.35	0.33	0.002	0.15
CV%		1.05	12.94	3.27	1.01	1.32	3.34

**p < 0.01, compared to control.

**Table 2:** Mean and standard error (SE), coefficient of variation (CV), relative coefficient of variation (CVt/CVnt)^1^ and amplitude of variation for six quantitative traits in the M_3_ generation of Rama, SI 1666 and IC 21706.

Character	Treatment	Dose	Mean ± SE				% of the control				CV%				CVt/CVnt				Amplitude of variation		
			Rama	SI 1666	IC 21706		Rama	SI 1666	IC 21706		Rama	SI 1666	IC 21706		Rama	SI 1666	IC 21706		Rama	SI 1666	IC 21706
	Control	Control	86.19 ± 2.21	106.72 ± 1.38	107.05 ± 0.19		100.00	100.00	100.00		3.63	1.83	0.25		1.00	1.00	1.00		73-95	67-112	98-125
	γ-rays	200 Gy	87.34 ± 0.45	130.47 ± 0.66	112.35 ± 0.33		101.33	122.25	104.95		0.74	0.68	0.41		0.20	0.37	1.64		43-130	109-181	99-145
		400 Gy	98.60 ± 0.31	107.25 ± 0.86	110.95 ± 0.18		114.40	100.50	103.64		0.45	1.14	0.23		0.12	0.62	0.92		56-119	92-127	96-127
Plant height		600 Gy	93.47 ± 0.76	131.42 ± 0.84	103.11 ± 0.50		108.45	123.14	96.32		1.16	0.90	0.69		0.32	0.49	2.76		73-114	105-169	62-124
	EMS	0.5%	93.61 ± 0.33	117.59 ± 0.47	111.63 ± 1.04		108.61	110.19	104.28		0.50	0.56	1.00		0.14	0.31	4.00		62-141	81-136	87-159
		1.0%	93.30 ± 0.56	109.92 ± 0.33	124.57 ± 0.88		108.25	103.00	116.37		0.85	0.42	1.32		0.23	0.23	5.28		63-147	64-168	86-148
		1.5%	94.41 ± 0.73	85.31 ± 0.46	111.52 ± 0.53		109.54	79.94	104.18		1.09	0.76	0.67		0.30	0.42	2.68		51-134	60-119	82-133
		2.0%	66.44 ± 9.49	92.35 ± 0.16	101.91 ± 0.50		77.09	86.53	95.20		21.16	0.24	0.70		5.83	0.13	2.80		73-135	79-110	62-129

	Control	Control	4.31 ± 0.26	3.94 ± 0.07	4.81 ± 0.06		100.00	100.00	100.00		8.62	2.49	1.66		1.00	1.00	1.00		1-5	0-6	0-6
	γ-rays	200 Gy	4.97 ± 0.13	9.62 ± 0.31	11.17 ± 0.13		115.31	244.16	232.22		3.66	4.52	1.45		0.42	1.82	0.87		0-29	2-18	8-22
Number of branches/plant		400 Gy	7.12 ± 0.21	8.66 ± 0.36	6.52 ± 0.33		165.20	219.80	135.55		4.23	5.88	7.18		0.49	2.36	4.33		2-33	3-13	2-12
		600 Gy	4.27 ± 0.18	4.68 ± 1.08	4.85± 0.23		99.07	118.78	100.83		5.83	32.68	6.60		0.68	13.12	3.98		2-6	2-9	2-12
	EMS	0.5%	6.44 ± 0.08	4.71 ± 0.25	6.88 ± 0.23		149.42	119.54	143.04		8.34	34.62	24.73		0.97	13.90	14.90		0-32	0-29	2-19
		1.0%	4.97 ± 0.14	7.64 ± 1.87	6.92 ± 0.17		115.31	193.91	143.87		3.99	7.61	3.55		0.46	3.06	2.14		1-13	4-18	0-12
		1.5%	8.62 ± 0.51	6.28 ± 0.19	8.00 ± 1.40		200.00	159.39	166.32		1.81	4.22	4.71		0.21	1.69	2.84		0-15	0-15	0-8
		2.0%	4.02 ± 0.14	3.45 ± 0.18	5.63 ± 0.37		93.27	87.56	170.05		5.04	7.50	9.23		0.58	3.01	5.56		2-21	0-7	0-7

	Control	Control	52.37 ± 0.24	51.07 ± 0.59	58.46 ± 0.29		100.00	100.00	100.00		0.64	1.69	0.70		1.00	1.00	1.00		46-84	21-63	24-55
Number of capsules/plant	γ-rays	200 Gy	66.36 ± 0.54	86.87 ± 0.72	88.18 ± 0.78		126.31	170.10	150.84		1.15	1.18	1.25		1.80	0.70	1.79		6-212	15-142	76-124
		400 Gy	66.32 ± 0.53	72.42 ± 0.45	57.37 ± 1.82		126.64	141.81	98.14		1.12	1.27	18.53		1.75	0.75	26.47		12-181	18-134	16-90
		600 Gy	45.26 ± 0.32	99.46 ± 0.89	48.99 ± 6.66		83.42	194.75	83.80		0.99	0.86	4.30		1.55	0.51	6.14		27-75	33-181	5-91
	EMS	0.5%	71.66 ± 0.44	52.01 ± 0.51	121.47 ± 1.09		136.83	101.84	207.78		2.56	3.47	1.76		4.00	2.05	2.51		9-237	10-335	5-372
		1.0%	37.32 ± 0.67	50.94 ± 0.32	64.03 ± 0.83		71.26	99.75	109.53		0.87	0.87	1.27		1.36	0.51	1.81		4-82	17-58	51-226
		1.5%	75.18 ± 0.54	38.45 ± 0.71	62.89 ± 0.75		143.56	75.29	107.58		1.01	2.63	1.54		1.58	1.56	2.20		6-137	7-66	7-63
		2.0%	28.69 ± 0.34	58.57 ± 0.32	56.18 ± 0.46		54.78	114.69	96.10		1.66	0.88	1.12		2.59	0.52	1.60		25-138	12-86	12-98

	Control	Control	59.09 ± 0.08	55.96 ± 0.26	65.03 ± 0.19		100.00	100.00	100.00		0.20	0.65	0.42		1.00	1.00	1.00		48-63	50-63	56-58
	γ-rays	200 Gy	56.47± 1.51	55.27 ± 0.38	57.94 ± 0.20		95.57	98.77	89.10		3.78	0.92	0.48		18.90	1.42	1.14		38-64	38-66	49-64
Number of seeds/ capsule		400 Gy	55.41± 0.16	55.71 ± 0.64	55.18 ± 0.10		93.77	99.55	84.85		0.40	1.62	0.46		2.00	2.49	1.10		40-64	48-62	50-61
		600 Gy	57.28 ± 0.26	59.87 ± 0.16	56.98 ± 0.45		96.94	106.99	87.62		0.65	0.37	1.12		3.25	0.57	2.66		44-62	52-64	48-60
	EMS	0.5%	58.01± 0.26	56.72 ± 0.12	59.34 ± 0.20		98.17	101.36	91.25		0.63	0.31	0.47		3.15	0.48	1.12		32-66	38-67	40-71
		1.0%	51.48 ± 0.10	55.90 ± 0.11	59.74 ± 0.24		87.12	99.89	91.87		0.27	0.28	0.86		1.35	0.43	2.05		40-64	48-63	44-64
		1.5%	58.36 ± 0.36	54.26 ± 0.27	58.98 ± 0.18		98.76	96.96	90.70		0.87	0.71	0.44		4.35	1.09	1.05		48-65	40-65	45-62
		2.0%	53.57± 0.39	54.22 ± 0.18	55.25 ± 0.34		90.66	96.89	84.96		1.08	0.47	0.58		5.40	0.72	1.38		48-64	44-56	48-64

	Control	Control	3.02 ± 0.01	3.28 ± 0.01	3.27 ± 0.01		100.00	100.00	100.00		0.42	0.16	0.63		1.00	1.00	1.00		2.85-3.27	2.74-3.48	2.81-3.16
	γ-rays	200 Gy	3.04 ± 0.01	3.21 ± 0.01	3.04 ± 0.02		100.66	97.87	92.97		0.20	0.22	0.17		0.48	1.38	0.27		2.90-3.69	2.81-3.65	2.85-3.19
1000-seed weight		400 Gy	3.12 ± 0.01	3.05 ± 0.02	3.03 ± 0.01		103.31	92.99	92.66		0.12	0.76	0.75		0.29	4.75	1.19		2.97-3.60	2.86-3.26	2.75-3.13
		600 Gy	3.11 ± 0.01	3.10 ± 0.01	2.97 ± 0.01		102.98	94.51	90.83		0.15	0.16	0.22		0.36	1.00	0.35		2.74-3.41	2.85-3.26	2.60-3.24
	EMS	0.5%	3.07 ± 0.01	3.12 ± 0.01	3.07 ± 0.01		101.66	95.12	93.88		0.33	0.13	0.31		0.79	0.81	0.49		2.08-3.81	2.06-3.66	2.10-3.24
		1.0%	3.13 ± 0.01	3.11 ± 0.01	3.05 ± 0.01		103.64	94.82	93.27		0.20	0.14	0.34		0.48	0.88	0.54		2.72-3.64	2.39-3.35	2.76-3.33
		1.5%	3.16 ± 0.01	3.04 ± 0.01	3.11 ± 0.01		104.64	92.68	95.11		0.07	0.17	0.12		0.17	1.06	0.19		2.74-3.60	2.28-3.52	2.25-3.18
		2.0%	3.00 ± 0.01	3.04 ± 0.01	3.09 ± 0.01		99.34	92.68	94.50		0.44	0.12	0.17		1.05	0.75	0.27		2.81-3.65	2.81-3.27	2.46-3.50
	Control	Control	9.31 ± 0.18	9.37 ± 0.13	12.42± 0.11		100.00	100.00	100.00		2.76	2.00	1.26		1.00	1.00	1.00		8.13-12.56	3.75-12.08	5.14-13.29
	γ-rays	200 Gy	11.62 ± 0.23	15.62 ± 0.14	15.63 ± 0.17		124.81	166.70	125.85		2.83	1.20	1.52		1.03	0.60	1.21		7.84-20.25	2.04-22.92	11.18-22.15
		400 Gy	11.19 ± 0.10	12.60 ± 0.21	9.58 ± 0.14		120.19	134.47	77.13		1.24	2.31	18.72		0.45	1.16	14.86		5.70-27.39	2.75-22.17	2.55-15.51
		600 Gy	8.20 ± 0.07	18.88 ± 0.17	8.14 ± 0.25		88.08	201.49	65.54		1.17	1.29	0.19		0.42	0.65	0.15		5.15-12.64	5.35-36.66	0.97-15.14
Seed yield/plant		0.5%	6.25 ± 0.09	8.90± 0.25	22.72 ± 1.13		67.13	94.98	182.93		1.98	2.05	3.63		0.72	1.03	2.88		4.10-26.73	6.92-38.90	7.48-41.79
	EMS	1.0%	13.22 ± 0.11	15.20 ± 0.10	11.75 ± 0.16		142.00	162.22	94.61		1.18	0.85	1.57		0.43	0.43	1.25		5.41-22.17	4.44-32.95	7.61-28.41
		1.5%	14.54 ± 0.09	6.38 ± 0.13	11.47± 0.32		156.18	68.09	92.35		0.89	0.91	1.89		0.32	0.46	1.50		1.21-15.66	3.85-16.84	4.71-29.16
		2.0%	4.79 ± 0.06	5.31 ± 0.05	9.67 ± 0.09		51.45	56.67	77.86		1.70	1.11	1.32		0.62	0.56	1.05		5.18-14.15	2.26-11.04	4.72-19.80

^1^CVt = coefficient of variation among plants from the mutagenic treatment; CVnt = coefficient of variation among plants from the control treatment.

**Table 3:** Phenotypic variance, residual variance, F-test (Vt/Vnt)^1^ and variance increase due to the mutagenic treatment (Dt)^2^, estimated for six quantitative characters in the M_3_ generation of Rama, SI 1666 and IC 21706 treated with γ-rays and EMS.

Character	Treatment	Dose	Phenotypic variance				Residual variance				“F” = Vt/Vnt				Dt		
			Rama	SI 1666	IC 21706		Rama	SI 1666	IC 21706		Rama	SI 1666	IC 21706		Rama	SI 1666	IC 21706
	Control	Control	37.92	3.16	2.30		9.77	3.80	0.07		1.00	1.00	1.00		0.00	0.00	0.00
	γ-rays	200 Gy	52.51	593.22	70.68		0.41	0.86	0.21		1.38	187.73**	30.73**		-9.36	-2.94	0.14
		400 Gy	186.27	110.32	148.45		0.19	1.50	0.07		4.91*	34.91**	64.54**		-9.58	-2.30	0.00
Plant		600 Gy	213.62	457.30	329.32		1.17	0.40	0.50		5.63**	144.72**	143.18**		-8.60	-3.40	0.43
height	EMS	0.5%	185.05	745.27	258.62		0.22	0.44	2.18		4.88*	235.84**	112.44**		-9.55	-3.36	2.11
		1.0%	295.34	162.14	492.68		0.63	0.22	1.56		7.79**	51.31**	214.21**		-9.14	-3.58	1.49
		1.5%	347.19	144.99	111.40		1.07	0.42	0.56		9.16**	45.88**	48.43**		-8.70	-3.38	0.49
		2.0%	461.20	28.62	156.78		180.29	0.05	0.51		12.16**	9.06**	68.17**		170.52	-3.75	0.44

	Control	Control	0.55	0.07	0.03		0.14	0.01	0.01		1.00	1.00	1.00		0.00	0.00	0.00
	γ-rays	200 Gy	1.79	12.61	16.75		0.03	0.19	0.03		3.25*	180.14**	558.33**		-0.11	0.18	0.02
Number		400 Gy	62.46	18.67	14.57		0.09	0.26	0.22		113.56**	266.71**	485.67**		-0.05	0.25	0.21
of		600 Gy	1.04	5.89	8.95		0.06	2.34	0.10		1.89	84.14**	298.33**		-0.08	2.33	0.09
branches/	EMS	0.5%	15.45	5.14	20.79		0.01	0.13	0.11		28.09**	73.43**	693.00**		-0.13	0.12	0.10
plant		1.0%	2.28	23.28	15.92		0.04	7.00	0.06		4.15*	332.57**	530.67**		-0.10	6.99	0.05
		1.5%	27.39	27.80	32.42		0.52	0.07	3.91		49.80**	397.14**	1080.67**		0.38	0.06	3.90
		2.0%	9.96	0.91	6.49		0.04	0.07	0.27		18.11**	13.00**	216.33**		-0.10	0.06	0.26

	Control	Control	8.35	7.46	2.35		0.11	0.71	0.17		1.00	1.00	1.00		0.00	0.00	0.00
Number	γ-rays	200 Gy	314.04	1190.50	177.33		0.59	1.05	1.21		37.61**	159.58**	75.46**		0.48	0.34	1.04
of		400 Gy	2007.43	1737.06	949.88		0.55	0.40	6.65		240.41**	232.85**	404.20**		0.44	-0.31	6.48
capsules/		600 Gy	108.37	2325.92	789.71		0.20	1.60	88.66		12.98**	311.79**	336.05**		0.09	0.89	88.49
plant	EMS	0.5%	1199.08	3844.48	4185.70		0.39	0.52	2.37		143.60**	515.35**	1781.15**		0.28	-0.19	2.20
		1.0%	989.44	349.98	212.55		0.91	0.21	1.38		118.50**	46.91**	90.45**		0.80	-0.50	1.21
		1.5%	994.33	335.57	2300.56		0.58	1.02	1.11		119.08**	44.98**	978.96**		0.47	0.31	0.94
		2.0%	482.33	95.01	568.55		0.23	0.20	0.42		57.76**	12.74**	241.94**		0.12	-0.51	0.25

	Control	Control	1.28	1.21	0.84		0.14	0.13	0.08		1.00	1.00	1.00		0.00	0.00	0.00
	γ-rays	200 Gy	18.68	4.05	22.51		4.54	0.28	0.08		14.59**	3.35*	26.80**		4.40	0.15	0.00
Number		400 Gy	26.32	13.85	18.53		0.05	0.82	0.02		20.56**	11.45**	22.06**		-0.09	0.69	-0.06
of		600 Gy	28.92	18.94	25.33		0.14	0.05	0.40		22.59**	15.65**	30.15**		0.00	-0.08	0.32
seeds/	EMS	0.5%	46.37	18.75	12.52		0.13	0.30	0.08		36.23**	15.50**	14.90**		-0.01	0.17	0.00
capsule		1.0%	46.93	15.99	12.35		0.02	0.02	0.12		36.66**	13.21**	14.70**		-0.12	-0.11	0.04
		1.5%	47.58	14.89	8.46		0.26	0.15	0.07		37.17**	12.31**	10.07**		0.12	0.02	-0.01
		2.0%	34.45	9.86	14.41		0.31	0.07	0.23		26.91**	8.15**	17.15**		0.17	-0.06	0.15

	Control	Control	0.01	0.01	0.00		0.00	0.00	0.00		1.00	1.00	1.00		0.00	0.00	0.00
	γ-rays	200 Gy	0.02	0.03	0.02		0.00	0.00	0.00		2.00	3.00	0.02		0.00	0.00	0.00
1000-		400 Gy	0.03	0.02	0.01		0.00	0.00	0.00		3.00	2.00	0.01		0.00	0.00	0.00
seed		600 Gy	0.06	0.02	0.02		0.00	0.00	0.00		6.00**	2.00	0.02		0.00	0.00	0.00
weight	EMS	0.5%	0.02	0.03	0.02		0.00	0.00	0.00		2.00	3.00	0.02		0.00	0.00	0.00
		1.0%	50.00	0.04	0.03		0.00	0.00	0.00		5000.00**	4.00*	0.03		0.00	0.00	0.00
		1.5%	0.06	0.025	0.02		0.00	0.00	0.00		6.00**	2.50	0.02		0.00	0.00	0.00
		2.0%	0.03	0.03	0.03		0.00	0.00	0.00		3.00	3.00	0.03		0.00	0.00	0.00

	Control	Control	0.22	0.22	0.20		0.07	0.03	0.02		1.00	1.00	1.00		0.00	0.00	0.00
	γ-rays	200 Gy	15.79	44.33	12.13		0.11	0.04	0.06		71.77**	201.50**	60.65**		0.04	0.01	0.04
Seed		400 Gy	45.36	57.26	15.65		0.02	0.09	0.08		206.18**	260.27**	78.25**		-0.05	0.05	0.06
yield/		600 Gy	6.74	103.02	19.67		0.01	0.06	2.53		30.64**	468.27**	98.35**		-0.06	0.03	2.51
plant	EMS	0.5%	58.15	163.06	168.82		0.02	0.02	0.13		264.32**	741.18**	844.10**		-0.05	-0.01	0.11
		1.0%	4.73	12.37	10.26		0.02	0.01	0.05		21.50**	56.23**	51.30**		-0.05	-0.02	0.03
		1.5%	53.38	9.97	75.81		0.02	0.04	0.20		242.64**	45.32**	379.05**		-0.05	0.01	0.18
		2.0%	16.27	1.00	20.85		0.01	0.00	0.02		73.95**	4.55*	104.25**		-0.06	-0.03	0.00

^1^Vt = variance among mutagen-treated plants; Vnt = variance among control plants. ^2^Dt = sr^2^ - so^2^, where sr^2^ = variance increase due to the mutagenic treatment, so^2^ = control residual variance. *^,^**p < 0.05 and p < 0.01, respectively, compared to control.
